# ﻿Two new brown rot polypores from tropical China

**DOI:** 10.3897/mycokeys.82.68299

**Published:** 2021-08-19

**Authors:** Meng Zhou, Chao-Ge Wang, Ying-Da Wu, Shun Liu, Yuan Yuan

**Affiliations:** 1 Institute of Microbiology, School of Ecology and Nature Conservation, Beijing Forestry University, Beijing 100083, China Beijing Forestry University Beijing China; 2 China Fire and Rescue Institute, Beijing 102202, China China Fire and Rescue Institute Beijing China

**Keywords:** Brown-rot fungi, multi-gene phylogeny, phylogeny, taxonomy

## Abstract

Brown-rot fungi are types of fungi that selectively degrade cellulose and hemicellulose from wood and are perhaps the most important agents involved in the degradation of wood products and dead wood in forest ecosystem. Two new brown-rot species, collected from southern China, are nested within the clades of *Fomitopsis* sensu stricto and *Oligoporus* sensu stricto, respectively. Their positions are strongly supported in the Maximum Likelihood phylogenetic tree of the concatenated the internal transcribed spacer (ITS) regions, the large subunit of nuclear ribosomal RNA gene (nLSU), the small subunit of nuclear ribosomal RNA gene (nuSSU), the small subunit of mitochondrial rRNA gene (mtSSU), the largest subunit of RNA polymerase II (RPB1), the second largest subunit of RNA polymerase II (RPB2) and the translation elongation factor 1-α gene (TEF1) sequences. *Fomitopsisbambusae*, only found on bamboo, is characterised by its resupinate to effused-reflexed or pileate basidiocarps, small pores (6–9 per mm), the absence of cystidia, short cylindrical to oblong-ellipsoid basidiospores measuring 4.2–6.1 × 2–2.3 μm. *Oligoporuspodocarpi* is characterised by white to pale cream pore surface, round or sometimes angular pores (5–6 per mm), broadly ellipsoid to reniform basidiospores measuring 3.8–4.2 × 2–2.3 μm and growing on *Podocarpus*. Illustrated descriptions of these two novel species, *Fomitopsisbambusae* and *Oligoporuspodocarpi*, are provided.

## ﻿Introduction

Wood-inhabiting basidiomycota can be grouped into two categories, white-rot and brown-rot fungi, according to their ability for decaying or decomposing wood. Brown-rot fungi selectively degrade cellulose and hemicellulose from wood and decayed material becomes reddish-brown or tan, crisp, causing massive cracks in the middle of a longitudinal crisscross. However, white-rot fungi can degrade all the components of wood and decayed material, become white or pale-yellow or light reddish-brown and expose the fibrous structure. The number of brown rot fungi is remarkably smaller compared to white rot fungi (Zhang 2003; Wu et al. 2020). Gilbertson (1981) has estimated that approximately 6% of the wood-rotting basidiomycetes in North America give a brown rot. On the other hand, Dai (2012) demonstrated that 14% of Chinese polypores in northern China can cause a brown rot (Cui et al. 2019). Brown-rot fungi are perhaps the most important agents involved in the degradation of wood products and in the degradation of dead wood in forest ecosystems. It is worth emphasising that the diversity of brown rot fungi is higher in high-latitude areas than in low-latitude areas and the number of brown rot fungi decreases from north to south in China (Zhou and Dai 2012; Dai et al. 2015), so that brown-rot fungi are infrequent in tropical areas.

As a cosmopolitan brown-rot genus of polypores, *Fomitopsis* P. Karst., was established by Karsten, based on *F.pinicola* (Sw.) P. Karst. (Karsten 1881). The genus was classified in the Fomitopsidaceae morphologically (Jülich 1981) and belonged to the *Antrodia* clade phylogenetically (Binder et al. 2005; Ortiz-Santana et al. 2013; Han et al. 2016). Han et al. (2016) confirmed that species, previously belonging to *Fomitopsis* sensu lato, were embedded in seven lineages and eleven species form the core group of *Fomitopsis*. In addition, four species *Fomitopsiscaribensis* B.K. Cui & Shun Liu, *F.eucalypticola* B.K. Cui & Shun Liu, *F.ginkgonis* B.K. Cui & Shun Liu and *F.roseoalba* A.M.S. Soares, Ryvarden & Gibertoni were introduced as new species and *F.bondartsevae* (Spirin) A.M.S. Soares & Gibertoni was proposed as a new combination (Soares et al. 2017; Tibpromma et al. 2017; Liu et al. 2019). In the latest study, ten species have been recognised in the *Fomitopsispinicola* complex (Haight et al. 2019; Liu et al. 2021). So far, 25 species have been accepted in *Fomitopsis* sensu stricto (s. str.).

*Oligoporus* Bref. (Polyporales, Basidiomycetes) was typified with *O.farinosus* Bref., 1888 (Syn. *O.rennyi* (Berk. & Broome) Kotl.) (Brefeld 1888). Recent phylogenetic analyses have demonstrated that *Oligoporus* and *Tyromyces* belong to different clades and that they were grouped within families Dacryobolaceae Jülich and Incrustoporiaceae Jülich (Binder et al. 2013; Floudas and Hibbett 2015; Justo et al. 2017). Shen et al. (2019) have proved *Oligoporus* s. str. is different from *Postia* s. str. in morphology and molecular phylogenetic analysis. Meanwhile, species in *Postia* s. str. have a broad host range growing both on angiosperm and gymnosperm wood, but *Oligoporus* s. str. grows only on gymnosperm wood (Donk 1971; Ryvarden and Melo 2014; Shen et al. 2019). So far, only two species have been accepted in *Oligoporus* s. str. (Shen et al. 2019).

During our investigations of brown-rot fungi in China, eight specimens were collected from Hainan Province in tropical China. Morphological examination shows these collections to represent two brown-rot polypores, corresponding to *Fomitopsis* s.s. and *Oligoporuss.s.* After phylogenetic analyses of the internal transcribed spacer (ITS) regions, the large subunit of nuclear ribosomal RNA gene (nLSU), the small subunit of nuclear ribosomal RNA gene (nuSSU), the small subunit of mitochondrial rRNA gene (mtSSU), the largest subunit of RNA polymerase II (RPB1), the second largest subunit of RNA polymerase II (RPB2) and the translation elongation factor 1-α gene (TEF1) sequences, two new species were confirmed as belonging to *Fomitopsis* s.s. and *Oligoporus* s.s.. In this paper, we describe and illustrate these two new species.

## ﻿Materials and methods

### ﻿Morphological studies

The examined specimens were deposited in the herbarium of the Institute of Microbiology, Beijing Forestry University (**BJFC**) in Beijing, China. Macro-morphological descriptions were based on the field notes and measurements of herbarium specimens. Colour terms followed Petersen (1996). Micro-morphological data were obtained from the dried specimens and observed under a light microscope following Chen et al. (2017) and Shen et al. (2019). Sections were studied at a magnification up to 1000× using a Nikon Eclipse 80i microscope with phase contrast illumination (Nikon, Tokyo, Japan). Drawings were made with the aid of a drawing tube. Microscopic features, measurements and drawings were made from slide preparations stained with Cotton Blue and Melzer’s Reagent. Spores were measured from sections cut from the tubes. To present the variation of spore size, 5% of measurements were excluded from each end of the range and are given in parentheses. The following abbreviations are used: IKI = Melzer’s Reagent, IKI– = neither amyloid nor dextrinoid, KOH = 5% potassium hydroxide, CB = Cotton Blue, CB– = acyanophilous, L = mean spore length (arithmetic average of all spores), W = mean spore width (arithmetic average of all spores), Q = variation in the L/W ratios between the specimens studied, n (a/b) = number of basidiospores (a) measured from given number (b) of specimens.

### ﻿DNA extraction and sequencing

A cetyltrimethylammonium bromide rapid plant genome extraction kit (Aidlab Biotechnologies Co., Ltd, Beijing, China) was used to extract the total genomic DNA from dried specimens according to the manufacturer’s instructions with some modifications (Song and Cui 2017; Xing et al. 2018). The ITS regions were amplified with the primer pairs ITS5 (GGA AGT AAA AGT CGT AAC AAG G) and ITS4 (TCC TCC GCT TAT TGA TAT GC) (White et al. 1990). The nLSU regions were amplified with the primer pairs LR0R (ACC CGC TGA ACT TAA GC) and LR7 (TAC TAC CAC CAA GAT CT) (http://www.biology.duke.edu/fungi/mycolab/primers.htm). The nuSSU regions were amplified with the primer pairs NS1(CCG GAG AGG GAG CCT GAG AAA C) and NS4 (CCC GTG TTG AGT CAA ATT A) (White et al. 1990). The mtSSU regions were amplified with the primer pairs MS1 (CAG CAG TCA AGA ATA TTA GTC AAT G) and MS2 (GCG GAT TAT CGA ATT AAA TAA C) (White et al. 1990). RPB1 was amplified with the primer pairs RPB1-Af (GAR TGY CCD GGD CAY TTY GG) and RPB1-Cr (CCN GCD ATN TCR TTR TCC ATR TA) (Matheny et al. 2002). RPB2 was amplified with the primer pairs fRPB2-5F (GAY GAY MGW GAT CAY TTY GG) and fRPB2-7CR (CCC ATR GCT TGY TTR CCC AT) (Matheny 2005). TEF1 was amplified with the primer pairs EF1-983F (GCY CCY GGH CAY CGT GAY TTY AT) and EF1-1567R (ACH GTR CCR ATA CCA CCR ATC TT) (Rehner and Buckley 2005). The PCR procedure followed that of Liu et al. (2019). The PCR products were purified with a Gel Extraction and PCR Purification Combo Kit (Spin-column) in Beijing Genomics Institute, Beijing, P.R. China. The purified products were then sequenced on an ABI-3730-XL DNA Analyzer (Applied Biosystems, Foster City, CA, USA) using the same primers as in the original PCR amplifications. The sequence quality was checked following Nilsson et al. (2012). All newly-generated sequences were submitted to GenBank and were listed in Tables 1 and 2.

**Table 1.** A list of species, specimens and GenBank accession numbers of sequences used in the phylogeny of *Fomitopsis*.

Species	Sample no.	GenBank accessions						References
		ITS	nLSU	nuSSU	mtSSU	tef1	rpb2	
* Antrodia heteromorpha *	Dai 12755	KP715306	KP715322	KR605908	KR606009	KP715336	KR610828	Chen and Cui (2015)
* Antrodia serpens *	Dai 14850	MG787582	MG787624	MG787731	MG787674	MG787849	MG787798	Chen et al. (2018)
* Buglossoporus quercinus *	JV 1406/1	KR605801	KR605740	KR605899	KR606002	KR610730	KR610820	Han et al. (2016)
* Buglossoporus quercinus *	LY BR 2030	KR605799	KR605738	KR605897	KR606000	KR610728	KR610818	Han et al. (2016)
* Daedalea quercina *	Dai 2260	KR605792	KR605731	KR605885	KR605988	KR610718	KR610808	Han et al. (2016)
* Daedalea quercina *	Dai 12659	KP171208	KP171230	KR605887	KR605990	KR610719	KR610810	Han et al. (2015)
*** Fomitopsis bambusae ***	**Dai 22110**	**MW937874**	**MW937881**	**MW937867**	**MW937888**	**MZ082980**	**MZ082974**	**Present study**
*** Fomitopsis bambusae ***	**Dai 22114**	**MW937875**	**MW937882**	**MW937868**	**MW937889**	**MZ082981**	**MZ082975**	**Present study**
*** Fomitopsis bambusae ***	**Dai 22116**	**MW937876**	**MW937883**	**MW937869**	**MW937890**	—	—	**Present study**
*** Fomitopsis bambusae ***	**Dai 21942**	**MW937873**	**MW937880**	**MW937866**	**MW937887**	**MZ082979**	—	**Present study**
* Fomitopsis betulina *	Cui 10756	KR605797	KR605736	KR605894	KR605997	KR610725	KR610815	Han et al. (2016)
* Fomitopsis betulina *	Dai 11449	KR605798	KR605737	KR605895	KR605998	KR610726	KR610816	Han et al. (2016)
* Fomitopsis bondartsevae *	X 1207	JQ700277	JQ700277	—	—	—	—	Soares et al. (2017)
* Fomitopsis bondartsevae *	X 1059	JQ700275	JQ700275	—	—	—	—	Soares et al. (2017)
* Fomitopsis cana *	Cui 6239	JX435777	JX435775	KR605826	KR605934	KR610661	KR610761	Li et al. (2013)
* Fomitopsis cana *	Dai 9611	JX435776	JX435774	KR605825	KR605933	KR610660	KR610762	Li et al. (2013)
* Fomitopsis caribensis *	Cui 16871	MK852559	MK860108	MK860124	MK860116	MK900482	MK900474	Liu et al. (2019)
* Fomitopsis durescens *	Overholts 4215	KF937293	KF937295	KR605835	KR605941	—	—	Han et al. (2014)
* Fomitopsis durescens *	O 10796	KF937292	KF937294	KR605834	KR605940	KR610669	KR610766	Han et al. (2014)
* Fomitopsis eucalypticola *	Cui 16594	MK852560	MK860110	MK860126	MK860118	MK900483	MK900476	Liu et al. (2019)
* Fomitopsis eucalypticola *	Cui 16598	MK852562	MK860113	MK860129	MK860121	MK900484	MK900479	Liu et al. (2019)
* Fomitopsis ginkgonis *	Cui 17170	MK852563	MK860114	MK860130	MK860122	MK900485	MK900480	Liu et al. (2019)
* Fomitopsis ginkgonis *	Cui 17171	MK852564	MK860115	MK860131	MK860123	MK900486	MK900481	Liu et al. (2019)
* Fomitopsis hemitephra *	O 10808	KR605770	KR605709	KR605841	KR605947	KR610675	—	Han et al. (2016)
* Fomitopsis iberica *	O 10810	KR605771	KR605710	KR605842	KR605948	KR610676	KR610771	Han et al. (2016)
* Fomitopsis iberica *	O 10811	KR605772	KR605711	KR605843	—	KR610677	KR610772	Han et al. (2016)
* Fomitopsis meliae *	Dai 10035	KR605774	KR605713	KR605847	KR605952	KR610683	—	Han et al. (2016)
* Fomitopsis meliae *	Ryvarden 16893	KR605776	KR605715	KR605849	KR605954	KR610681	KR610775	Han et al. (2016)
* Fomitopsis mounceae *	DR-366	KF169624	—	—	—	KF178349	KF169693	Haight et al. (2019)
* Fomitopsis mounceae *	JAG-08-19	KF169626	—	—	—	KF178351	KF169695	Haight et al. (2019)
* Fomitopsis nivosa *	JV 0509/52 X	KR605779	KR605718	KR605853	KR605957	KR610686	KR610777	Han et al. (2016)
* Fomitopsis nivosa *	Man 09	MF589766	MF590166	—	—	—	—	Liu et al. (2019)
* Fomitopsis ochracea *	ss5	KF169609	—	—	—	KF178334	KF169678	Haight et al. (2016)
* Fomitopsis ochracea *	ss7	KF169610	—	—	—	KF178335	KF169679	Haight et al. (2016)
* Fomitopsis ostreiformis *	IRET 22	KY449363	—	—	—	—	—	Thangamalai et al. (2018)
* Fomitopsis ostreiformis *	LDCMY 21	KY111252	—	—	—	—	—	Thangamalai et al. (2018)
* Fomitopsis palustris *	Cui 7597	KP171213	KP171236	KR605854	KR605958	KR610687	KR610778	Han et al. (2015)
* Fomitopsis palustris *	Cui 7615	KR605780	KR605719	KR605855	KR605959	KR610688	KR610779	Han et al. (2015)
* Fomitopsis pinicola *	Cui 10532	KP171214	KP171237	KR605858	KR605962	KR610691	KR610782	Han et al. (2015)
* Fomitopsis pinicola *	Cui 10312	KR605781	KR605720	KR605856	KR605960	KR610689	KR610780	Han et al. (2016)
* Fomitopsis roseoalba *	AS 1496	KT189139	KT189141	—	—	—	—	Tibpromma et al. (2017)
* Fomitopsis roseoalba *	AS 1566	KT189140	KT189142	—	—	—	—	Tibpromma et al. (2017)
* Fomitopsis schrenkii *	JEH-144	KF169621	—	—	—	MK236355	MK208857	Haight et al. (2019)
* Fomitopsis schrenkii *	JEH-150	KF169622	—	—	—	MK236356	MK208858	Haight et al. (2019)
* Fomitopsis subtropica *	Cui 10154	JQ067652	JX435773	—	—	—	—	Li et al. (2013)
* Fomitopsis subtropica *	Cui 10578	KR605787	KR605726	KR605867	KR605971	KR610698	KR610791	Han et al. (2016)
* Laetiporus zonatus *	Dai 13633	KX354481	KX354508	KX354547	KX354589	KX354635	KX354676	Jie and Cui (2017)
* Laetiporus zonatus *	Cui 10404	KF951283	KF951308	KX354551	KX354593	KX354639	KT894797	Jie and Cui (2017)
* Niveoporofomes spraguei *	JV 0509/62	KR605786	KR605725	KR605864	KR605968	KR610697	KR610788	Han et al. (2016)
* Niveoporofomes spraguei *	4638	KR605784	KR605723	KR605862	KR605966	KR610696	KR610786	Han et al. (2016)
* Rhodofomes rosea *	Cui 10633	KR605782	KR605721	KR605860	KR605964	KR610693	KR610784	Han et al. (2016)
* Rhodofomes rosea *	JV 1110/9	KR605783	KR605722	KR605861	KR605965	KR610694	KR610785	Han et al. (2016)
* Rhodofomitopsis feei *	Ryvarden 37603	KC844850	KC844855	KR605838	KR605944	KR610670	KR610768	Han and Cui (2015)
* Rhodofomitopsis feei *	Oinonen 6011906	KC844851	KC844856	KR605837	KR605943	KR610671	KR610767	Han and Cui (2015)
* Rubellofomes cystidiatus *	Cui 5481	KF937288	KF937291	KR605832	KR605938	KR610667	KR610765	Han et al. (2014)
* Rubellofomes cystidiatus *	Yuan 6304	KR605769	KR605708	KR605833	KR605939	KR610668	—	Han et al. (2016)

**Table 2.** A list of species, specimens and GenBank accession numbers of sequences used in the phylogeny of *Oligoporus*.

Species	Sample no.	GenBank accessions							References
		ITS	nLSU	nuSSU	mtSSU	TEF1	RPB2	RPB1	
* Amaropostia stiptica *	Cui 10043	KX900906	KX900976	KX901119	KX901046		KX901219	KX901167	Shen et al. (2019)
* Amaropostia stiptica *	Cui 10981	KX900907	KX900977	KX901120	KX901047		KX901220	KX901168	Shen et al. (2019)
* Amylocystis lapponica *	HHB-13400	KC585237	KC585059						Ortiz-Santana et al. (2013)
* Amylocystis lapponica *	OKM-4118	KC585238	KC585060						Ortiz-Santana et al. (2013)
* Antrodia serpens *	Dai 7465	KR605813	KR605752	KR605913	KR606013	KR610742	KR610832		Han et al. (2016)
* Antrodia serpens *	Dai 14850	MG787582	MG787624	MG787731	MG787674	MG787849	MG787798		Chen et al. (2018)
* Calcipostia guttulata *	Cui 10028	KF727433	KJ684979	KX901139	KX901066	KX901277	KX901237	KX901182	Shen et al. (2019)
* Calcipostia guttulata *	KHL 11739(GB)	EU118650	EU118650						Larsson direct submission
* Cyanosporus caesius *	Dai 12605	KX900883	KX900953	KX901096	KX901021		KX901206	KX901159	Shen et al. (2019)
* Cyanosporus caesius *	Dai 12974	KX900884	KX900954	KX901097	KX901022	KX901258	KX901207	KX901160	Shen et al. (2019)
* Cyanosporus subcaesius *	KA12-1375	KR673585							Kim et al. (2015)
* Cyanosporus subcaesius *	K(M)32713	AY599576							Yao et al. (2005)
* Cystidiopostia hibernica *	Cui 2658	KX900905	KX900975	KX901118	KX901045		KX901218		Shen et al. (2019)
* Cystidiopostia hibernica *	K(M)17352	AJ006665							Yao et al. (2005)
* Cystidiopostia pileata *	Cui 5721	KF699127	KX900960	KX901121	KX901049	KX901268	KX901221	KX901169	Shen et al. (2019)
* Cystidiopostia pileata *	Cui 10034	KX900908	KX900956	KX901122	KX901050	KX901269	KX901222	KX901170	Shen et al. (2019)
* Fuscopostia duplicata *	Cui 10366	KF699124	KJ684975	KR605927	KR606026	KR610755	KR610844	KX901173	Han et al. (2016)
* Fuscopostia duplicata *	Dai 13411	KF699125	KJ684976	KR605928	KR606027	KR610756	KR610845	KX901174	Han et al. (2016)
* Fuscopostia fragilis *	Cui 10020	KX900912	KX900982	KX901126	KX901054	KX901270	KX901226		Shen et al. (2019)
* Fuscopostia fragilis *	Cui 10088	KF699120	KJ684977	KX901127	KT893749		KT893745		Han et al. (2016)
*** Oligoporus podocarpi ***	**Dai22042**	**MW93787777**	**MW937884**	**MW937870**	**MW937891**	**MZ082982**	**MZ082976**	**MZ005579**	**Present study**
*** Oligoporus podocarpi ***	**Dai22043**	**MW937878**	**MW937885**	**MW937871**	**MW937892**	**MZ082983**	**MZ082977**	**MZ005580**	**Present study**
*** Oligoporus podocarpi ***	**Dai22044**	**MW937879**	**MW937886**	**MW937872**	**MW937893**	**MZ082984**	**MZ082978**	**MZ005581**	**Present study**
* Oligoporus rennyi *	KEW 57	AY218416	AF287876						Ortiz-Santana et al. (2013)
* Oligoporus rennyi *	MR 10497	JX090117							Ortiz-Santana et al. (2013)
* Oligoporus sericeomollis *	Cui 9560	KX900919	KX900989	KX901140	KX901067			KX901183	Shen et al.(2019)
* Oligoporus sericeomollis *	Cui 9870	KX900920	KX900990	KX901141	KX901068			KX901184	Shen et al. (2019)
* Osteina obducta *	Cui 9959	KX900923	KX900993	KX901143	KX901070		KX901239		Shen et al. (2019)
* Osteina obducta *	Cui 10074	KX900924	KX900994	KX901144	KX901071		KX901240		Shen et al. (2019)
* Osteina undosa *	Dai 7105	KX900921	KX900991	KX901142	KX901069		KX901238		Shen et al. (2019)
* Osteina undosa *	L-10830	KC585396	KC585229						Ortiz-Santana et al. (2013)
* Postia hirsuta *	Cui 11180	KJ684971	KJ684985		KX901039				Shen and Cui (2014)
* Postia hirsuta *	Cui 11237	kj684970	KJ684984	KX901113	KX901038	KX901266			Shen and Cui (2014)
* Postia lactea *	Cui 9319	KX900894	KX900964	KX901106	KX901031	KX901262	KX901213	KX901165	Shen et al. (2019)
* Postia lactea *	Cui 11511	KX900893	KX900963	KX901105	KX901030	KX901261	KX901212	KX901164	Shen et al. (2019)
* Postia lowei *	Cui 9585	KX900898	KX900968	KX901110	KX901035				Shen et al. (2019)
* Postia lowei *	X1373	KC595941							Ortiz-Santana et al. (2013)
* Postia ochraceoalba *	Cui 10802	KM107903	KM107908	KX901115	KX901041		KX901216		Shen et al. (2015)
* Postia ochraceoalba *	Cui 10825	KM107902	KM107907	KX901114	KX901040		KX901215		Shen et al. (2015)
* Spongious gloeoporus *	Cui 9507	KM107901	KM107906	KX901132	KX901059		KX901231		Shen et al. (2015)
* Spongious gloeoporus *	Cui 10401	KX900915	KX900985	KX901133	KX901060		KX901232		Shen et al. (2015)
* Spongiporus floriformis *	Cui 10292	KM107899	KM107904	KX901131	KX901058	KX901274	KX901230	KX901178	Shen et al. (2015)
* Spongiporus floriformis *	Dai 13887	KX900914	KX900984	KX901130	KX901057	KX901273	KX901229	KX901177	Shen et al. (2019)

### ﻿Phylogenetic analyses

**Figure 1.**
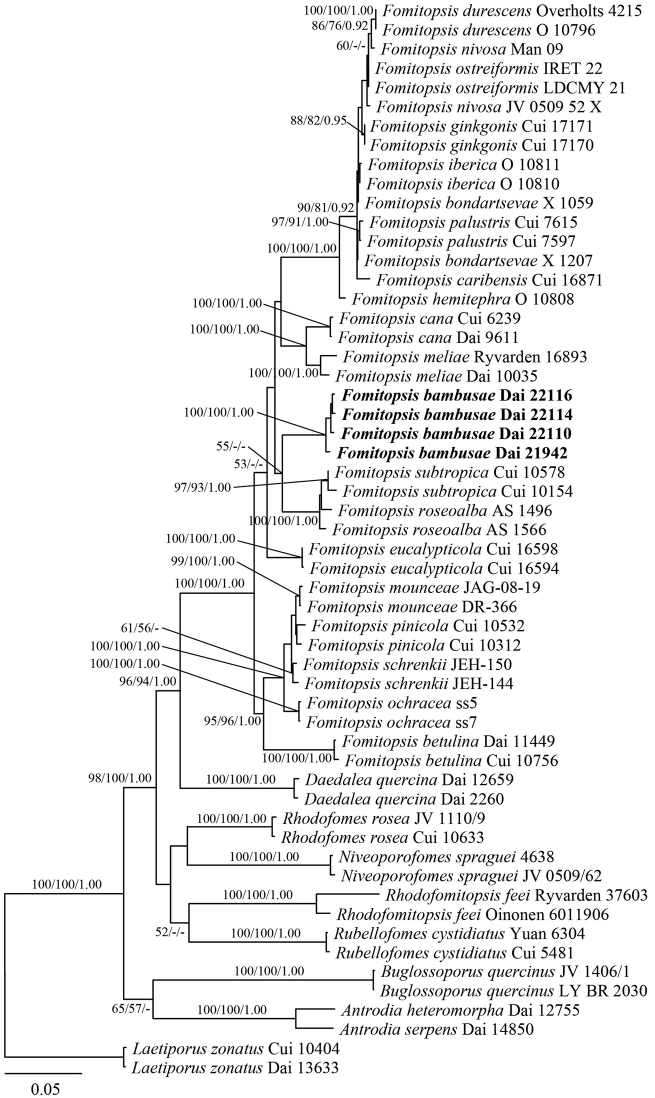
Maximum Likelihood phylogenetic tree of the new *Fomitopsis* species, based on multi-genes sequences data. Branches are labelled with bootstrap values (MP/ML) higher than 50% and posterior probabilities (BI) more than 0.90, respectively. Bold names: New species.

**Figure 2.**
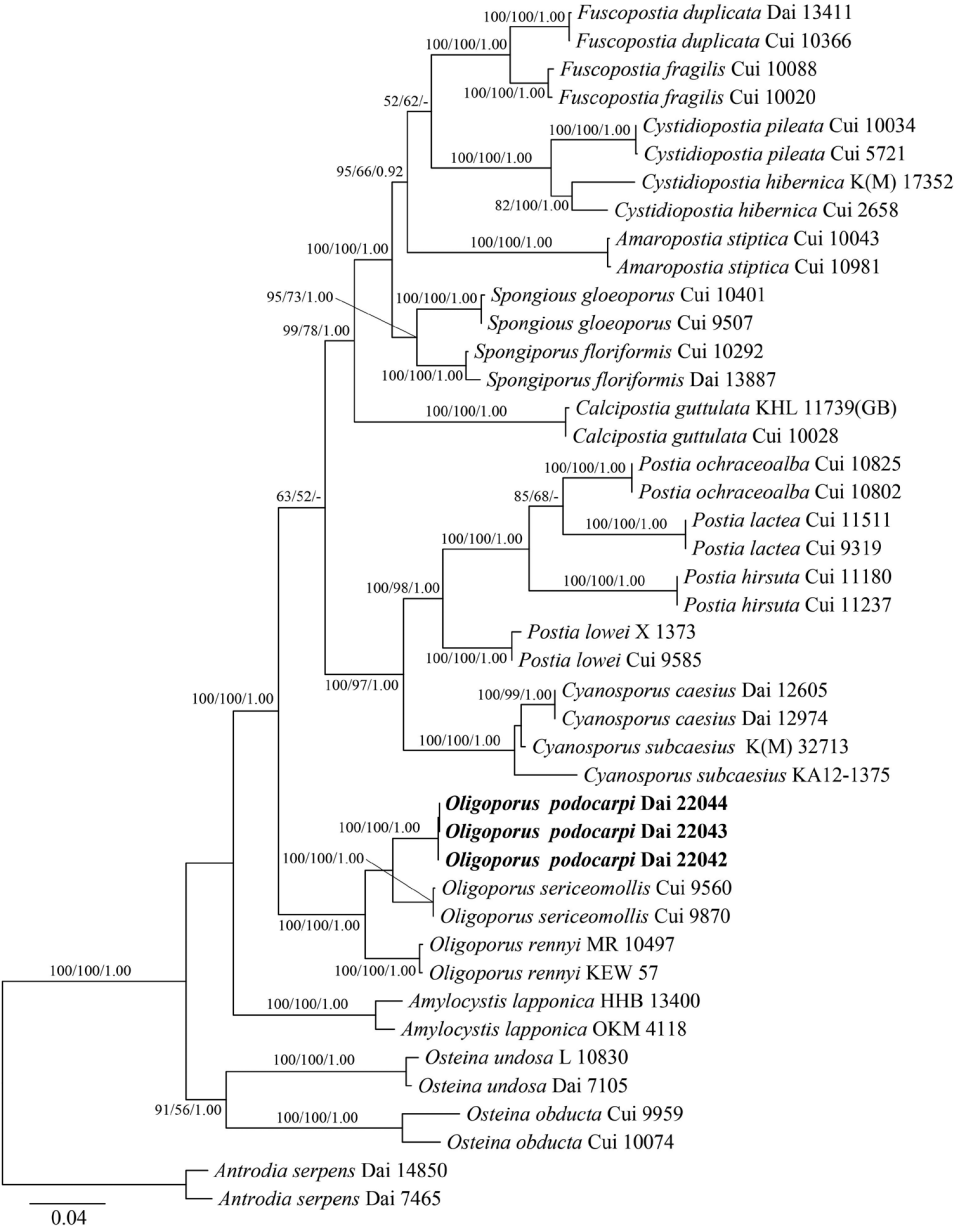
Maximum Likelihood phylogenetic tree of the new *Oligoporus* species, based on multi-genes sequences data. Branches are labelled with bootstrap values (MP/ML) higher than 50% and posterior probabilities (BI) more than 0.90, respectively. Bold names: New species.

New sequences, deposited in GenBank (http://www.ncbi.nlm.nih.gov/genbank/) (Table 1), were aligned with additional sequences retrieved from GenBank (Table 1) using BioEdit 7.0.5.3 (Hall 1999) and ClustalX 1.83 (Thompson et al. 1997), followed by manual adjustment. Sequence alignment was deposited at TreeBase (http://purl.org/phylo/treebase/; submission ID 28131). In phylogenetic reconstruction, sequences of *Laetiporuszonatus* B.K. Cui & J. Song, obtained from GenBank, were used as outgroups in the phylogeny of *Fomitopsis* (Fig. 1) while sequences of *Antrodiaserpens* (Fr.) P. Karst. were used as outgroups in the phylogeny of *Oligoporus* (Fig. 2).

Maximum Parsimony (MP) analysis was applied to those two phylogenies and trees construction procedure were performed in PAUP* version 4.0b10 (Swofford 2002). Settings for phylogenetic analyses in this study followed the approach of Zhu et al. (2019) and Song and Cui (2017). All characters were equally weighted and gaps were treated as missing data. Trees were inferred using the heuristic search option with TBR branch swapping and 1000 random sequence additions. Max-trees were set to 5000, branches of zero length were collapsed and all parsimonious trees were saved. Clade robustness was assessed using a bootstrap (BT) analysis with 1000 replicates (Felsenstein 1985). Descriptive tree statistics tree length (TL), consistency index (CI), retention index (RI), rescaled consistency index (RC) and homoplasy index (HI) were calculated for each Maximum Parsimonious Tree (MPT) generated.

Maximum Likelihood (ML) analysis was conducted with RAxML-HPC252 on Abe through the CIPRES Science Gateway (www.phylo.org) and involved 100 ML searches. All model parameters were estimated by the programme. Only the best Maximum Likelihood tree from all searches was kept. The Maximum Likelihood bootstrap values (ML-BS) were performed using a rapid bootstrapping with 1000 replicates. The phylogenetic tree was visualised using Treeview (Page 1996).

MrModeltest 2.3 (Posada and Crandall 1998; Nylander 2004) was used to determine the best-fit evolution model for two combined matrices to reconstruct phylogenetic analyses as a 6-gene dataset (ITS+nLSU+nuSSU+mtSSU+RPB2+TEF1) and a 7-gene dataset (ITS+nLSU+nuSSU+mtSSU+RPB1+RPB2+TEF1) for Bayesian Inference (BI). Bayesian Inference was calculated with MrBayes 3.2.6 (Ronquist et al. 2012), with a general time reversible (GTR) model of DNA substitution and a gamma distribution rate variation across sites. Four Markov chains were run for two runs from random starting trees for one million generations and trees were sampled every 100 generations. The burn-in was set to discard 25% of the trees. A majority rule consensus tree of all remaining trees was calculated. Branches that received bootstrap support for Maximum Parsimony (MP), Maximum Likelihood (ML) and Bayesian Posterior Probabilities (BPP) greater than or equal to 75% (MP and ML) and 0.95 (BPP) were considered as significantly supported.

## ﻿Results

### ﻿Molecular phylogeny

The phylogeny of *Fomitopsis*, based on a combined 6-gene (ITS, nLSU, nuSSU, mtSSU, RPB2, TEF1) dataset, included sequences from 64 fungal samples representing 29 taxa. They were downloaded from GenBank and generated in the present study (Table 1). The dataset had an aligned length of 4718 characters, including gaps (680 characters for ITS, 1343 characters for nLSU, 1013 characters for nuSSU, 547 characters for mtSSU, 648 characters for RPB2, 487 characters for TEF1), of which 3346 characters were constant, 1860 were variable and parsimony-uninformative, and 1212 were parsimony-informative. Maximum parsimony analysis yielded one equally-parsimonious tree (TL = 3802, CI = 0.544, RI = 0.787, RC = 0.428, HI = 0.456) and the MP tree is shown in Fig. 1. The best model for the combined ITS+nLSU+nuSSU+mtSSU+RPB2+TEF1 sequence dataset was estimated and applied in the Bayesian analysis was GTR+I+G with equal frequency of nucleotides, lset nst = 6 rates = invgamma; prset statefreqpr = dirichlet (1,1,1,1). Bayesian analysis resulted in a concordant topology with an average standard deviation of split frequencies = 0.008975.

The phylogeny of *Oligoporus*, combined 7-gene (ITS, nLSU, nuSSU, mtSSU, RPB1, RPB2, TEF1) dataset, included sequences from 43 fungal samples representing 21 taxa. They were downloaded from GenBank and generated in the present study (Table 2). The dataset had an aligned length of 5772 characters, including gaps (612 characters for ITS, 1302 characters for nLSU, 1009 characters for nuSSU, 491 characters for mtSSU, 1231 characters for RPB1, 648 characters for RPB2, 479 characters for TEF1), of which 4127 characters were constant, 129 were variable and parsimony-uninformative and 1516 were parsimony informative. Maximum parsimony analysis yielded four equally-parsimonious trees (TL = 3925, CI = 0.600, RI = 0.784, RC = 0.471, HI = 0.400) and a strict consensus tree of these trees is shown in Fig. 2. The best model for the combined ITS+nLSU+nuSSU+mtSSU+RPB1+RPB2+TEF1 sequence dataset was estimated and applied in the Bayesian analysis was GTR+I+G with equal frequency of nucleotides, lset nst = 6 rates = invgamma; prset statefreqpr = dirichlet (1,1,1,1). Bayesian analysis resulted in a concordant topology with an average standard deviation of split frequencies = 0.008567.

In our phylogenies (Figs 1 and 2), five samples on bamboo formed an independent lineage in the *Fomitopsis* s.s. clade with strong support (100% ML, 100% MP, 1.00 BPPs) and are distant from other taxa in the genus. Both morphology and rDNA sequence data confirmed that the five samples represent a new species in *Fomitopsis*. Meanwhile, three samples on *Podocarpus* were nested in the *Oligoporus* s.s. clade and formed an independent lineage with a robust support (100% ML, 100% MP, 1.00 BPPs). Both morphology and rDNA sequence data confirmed that the three samples represent a new species in *Oligoporus*.

**Table 3.** A comparison of species in the *Fomitopsis*.

Species	Holotype	Basidiocarps	Pileal surface	Pore surface	Pore (per mm.)	Hyphal system	Cystidia/cystidioles	Basidiospores	References
* F. abieticola *	China	Annual to perennial; pileate	Cream to pinkish buff	Cream to pinkish buff when fresh, becoming buff to curry-yellow when dry	Round to angular, 2–4	Trimitic	Cystidia absent; fusoid cystidioles occasionally present, 17.5–50.2 × 4.3–9.5 μm	Oblong-ellipsoid to ellipsoid, 7–9 × 4–5 µm.	Liu et al. (2021)
*** F. bambusae ***	**China**	**Annual, resupinate to effused-reflexed or pileate**	**Pluish grey when fresh, pale mouse-grey to greyish-sepia when dry**	**Bluish-grey to pale mouse-grey when fresh, becoming mouse-grey to dark grey when dry**	**Round to angular, 6–9**	**Dimitic**	**Cystidia absent; fusoid cystidioles present, 11–18 × 2.5–4 μm**	**Cylindrical to oblong ellipsoid, 4.2–6.1 × 2–2.3 µm**	**Present study**
* F. betulina *	Norway	Annual; pileate	Whitish to mouse-coloured or brownish	White to pale brownish	Round to angular, 3–5	Di-trimitic	Absent	Cylindrical, slightly allantoid, 5–6 × 1.5–1.7 µm.	Ryvarden and Melo (2014)
* F. bondartsevae *	Russia	Annual; effused-reflexed to pileate			Round to angular, 2–3	Trimitic	Cystidia absent; fusoid cystidioles present, 18–26 × 4.5–6 μm	Cylindrical, 6–7.2 × 2.2–2.5 µm.	Spirin (2002)
* F. cana *	China	Annual; resupinate to effused-reflexed or pileate	Pale mouse-grey to dark grey, azonate	Cream to straw coloured turning mouse-grey to dark grey	Angular, 5–8	Trimitic	Cystidia absent; fusoid cystidioles occasionally present, 9–16 × 3–5 μm	Cylindrical to oblong ellipsoid,5–6.2× 2.1–3 μm.	Li et al. (2013)
* F. caribensis *	Puerto Rico.	Annual; pileate, sessile	White to cream buff when fresh, cream buff to curry-yellow at base	White to cream when fresh, becoming cream to pinkish-buff when dry	Round to angular, 6–9	Dimitic	Cystidia absent; fusoid cystidioles occasional, hyaline, thin-walled, 12.5–23.5 × 2.5–4 μm	Cylindrical to oblong-ellipsoid, 6–7.5 × 2.3–3.1 μm.	Liu et al. (2019)
* F. durescens *	USA	Annual; sessile	Cream coloured to pale buff, drying tan	White to cream coloured, ochraceous on drying	Round to angular, 4–5	Trimitic	Cystidia absent; fusoid cystidioles present, 14–16 × 5–6 μm	Narrowly cylindrical, 6–8 × 1.5–2.5 µm	Gilbertson and Ryvarden (1986)
* F. eucalypticola *	Australia	Annual to biennial; effused-reflexed to pileate	Cream to salmon-coloured when young, straw yellow to clay-pink	Cream to yellow when fresh, buff to clay-buff when dry	Round to angular, 3–5	Trimitic	Cystidia absent; fusoid cystidioles occasionally present, 15–36 × 2–5.3 μm	Cylindrical to oblong-ellipsoid, 5.8–9.1 × 2.7–5 μm.	Liu et al. (2019)
* F. ginkgonis *	China	Annual; pileate, imbricate	Dirty greyish-brown to mouse-grey	Pinkish-buff to cinnamon-buff	Round to angular, 3–6	Trimitic	Cystidia absent; fusoid cystidioles occasionally present, 12.5–27.6 × 2.8–4.1 μm	Cylindrical, 7.2–9 × 2.2–3 μm.	Liu et al. (2019)
* F. hemitephra *	New Zealand	Perennial; solitary, attached by a broad lateral base	Tobacco brown or fuscous.	White or straw to isabelline	Round or slightly angular, 6–7	Trimitic	Cystidia absent; cystidioles, 6–8 × 3.5–4 µm	Elliptic-oblong, 4–6 × 2–2.5 μm.	Cunningham (1965)
* F. hengduanensis *	China	Annual to perennial; pileate	Pale dark grey to reddish-brown at base and cream to flesh-pink towards the margin	white to cream when fresh, becoming buff to straw-yellow	Round to angular, 6–8	Trimitic	Cystidia absent; fusoid cystidioles occasionally present, 13.2–36.5 × 2.5–5.4 μm	Oblong-ellipsoid to ellipsoid, 5.2–6 × 3.2–3.6 µm.	Liu et al. (2021)
* F. iberica *	Portugal	Annual; sessile, dimidiate, single or imbricate	White to cream when young. drying honey-coloured to brown	Pale, white, cream to straw-coloured	Round to ellipsoid, 3–4 per mm	Trimitic	Cystidia absent; pointed cystidioles present, 20–27 × 4–5–5 µm	Cylindrical to distinctly fusoid, 6–8 × 2.8–3.7 µm.	Melo and Ryvarden (1989)
* F. kesiyae *	Vietnam	Annual; pileate	Buff yellow to orange-yellow buff	White to cream when fresh, olivaceous buff to cinnamon-buff when dry	Round to angular, 6–8	Dimitic	Cystidia absent; fusoid cystidioles occasionally present, 11.5–30.4 × 2.6–6 μm	Oblong-ellipsoid to ellipsoid, 4.8–5.3 × 3–3.5 µm.	Liu et al. (2021)
* F. massoniana *	China	Annual; effused-reflexed to pileate	Buff-yellow to apricot-orange	White to cream when fresh, cream to buff	Round, 5–7	Dimitic	Cystidia absent; fusoid cystidioles occasionally present, 14.8–36 × 3.8–6 μm	Oblong-ellipsoid, 6.2–7.3 × 3.3–4 µm.	Liu et al. (2021)
* F. meliae *	USA	Annual or biennial; sessile, pilei single to imbricate, dimidiate	Ivory to tan or cinereous	Ochraceous	Round to angular, 5–7	Trimitic	Cystidia absent; fusoid cystidioles present, 15–23 × 4–5 µm	Cylindrical, slightly fusiform, tapering to the apex, 6–8 × 2.5–3 µm.	Gilbertson (1981)
* F. mounceae *	Canada	Perennial; pileate	Brownish-orange to black at base and pale orange to greyish-orange towards the margin	Yellowish-white, greyish-yellow, pale orange to light ochraceous buff, bright reddish-brown when dry	Round, 3–5	Dimitic	Cystidia obclavate to subfusiform with subacute or rounded apices, 16–35 × 3–6.5 µm	Ellipsoid to cylindrical, 5.8–6.6 × 3.4–4 µm.	Haight et al. (2019)
* F. nivosa *	Brazil	Annual to biennial; sessile, dimidiate, single to imbricate	Cream to pale sordid brown or tan	Cream to pale sordid brown or tan	Round to angular, 6–8	Trimitic	Cystidia absent; cystidioles broadly rounded, subapically contracted, 12–15 × 4–5 µm	Cylindrical, 6–9 – 2–3 µm	Gilbertson and Ryvarden (1986)
* F. ochracea *	Canada	Perennial; pileate	Brownish-grey to greyish-brown at base and orange white to pale orange towards the margin	Pale yellow, pale orange, light ochraceous buff, reddish-brown when dry	Round, 4–5	Trimitic	Cystidia absent; fusoid cystidioles occasionally present, 20–40 × 4–6.5 μm	Broadly ellipsoid, 5.1–5.9 × 3.6–4 µm.	Stokland and Ryvarden (2008); Haight et al. (2019)
* F. ostreiformis *	Singapore	Annual; sessile or effuse-reflexed	Greyish pileal surface	White or greyish-white	Round to angular, 3–4	Trimitic	Cystidia absent; cystidioles present, 10–17 × 2.8–4 μm	Cylindrical, 4.2–5.6 × 1.4–2.6 pm	De (1981); Hattori (2003)
* F. palustris *	USA	Perennial; sessile, horizontal, applanate	Dingy ochraceous to ochraceous buff, suffused dingy brownish-vinaceous	Vinaceous drab to brownish-vinaceous but pallid ochraceous near the margin	Angular, 7–9	Dimitic	absent	Cylindrical, 3.7–4.7 × 2–2.5 µm.	Corner (1989); Hattori (2003)
* F. pinicola *	Europe	Perennial; pileate	Brownish-orange to black at base and buff-yellow to cinnamon towards the margin	Cream coloured becoming citric yellow when bruised	Round, 4–6	Trimitic	Cystidia present, 18–90 × 3–9 μm	Cylindrical-ellipsoid, 6–9 × 3–4.5 µm.	Ryvarden and Melo (2014); Haight et al. (2019)
* F. roseoalba *	Brazil	Annual; pileate, resupinate to effused-reflexed	White to pink when fresh, cream to greyish when dry	White to cream when fresh and ochraceous when dried	Round to angular, 4–6	Trimitic	absent	Ellipsoid to sub-cylindrical, 3–4.9 × 1.8–2 µm.	Tibpromma et al. (2017)
* F. schrenkii *	USA	Perennial; effused-reflexed to pileate	Greyish-orange to olive brown at base and greyish-orange to greyish-yellow towards the margin	Pale yellow, pale orange, cream buff, reddish-brown when dry	Round, 3–4	Dimitic	Cystidia cylindrical, subulate, or subfusiform with subacute, 16–30 × 3–8 µm	Ellipsoid to broadly cylindrical, 5.7–6.7 × 3.7–4.2 µm.	Haight et al. (2019)
* F. subpinicola *	China	Annual; pileate	Apricot-orange, scarlet to fuscous	White to cream when fresh, turning buff yellow to buff when dry	Round, 6–8	Dimitic	Cystidia absent; fusoid cystidioles occasionally present, 14.5–34.6 × 3.2–7.2 μm	Oblong-ellipsoid to ellipsoid, 4.3–5.5 × 2.7–3.3 µm.	Liu et al. (2021)
* F. subtropica *	China	Annual; resupinate to effused-reflexed or pileate	Straw-yellow when young, becoming pale mouse-grey to flesh-pink with age.	Cream to straw coloured or pale pinkish	Angular, 6–9	Trimitic	Cystidia absent; fusoid cystidioles occasionally present, 9–15 × 3–4 μm	Cylindrical to oblong-ellipsoid, 3.2–4 × 1.8–2.1 µm.	Li and Cui (2013)
* F. tianshanensis *	China	Annual to perennial; effused-reflexed to pileate	Dark bluish-grey to yellowish-brown	Cream to pinkish-buff when fresh, becoming faint yellow to light pink when dry	Round to angular, 1–3	Dimitic	Cystidia absent; fusoid cystidioles occasionally present, 15.5–44 × 3.3–6.5 μm	Oblong-ellipsoid, 6.3–7 × 3.2–3.8 µm.	Liu et al. (2021)

### ﻿Taxonomy

#### ﻿ Fomitopsis bambusae

Taxon classificationFungiPolyporalesFomitopsidaceae

Y.C. Dai, Meng Zhou & Yuan Yuan sp. nov.

B08F1D81-E3C5-5DEE-AD3F-A6D9CEC9CCC7

MycoBank No: 839359

Figs 3 , 4

##### Diagnosis.

*Fomitopsisbambusae* is characterised by resupinate to effused-reflexed or pileate, soft corky basidiocarps with bluish-grey pores, small pores measuring 6–9 per mm, cylindrical to oblong ellipsoid basidiospores measuring 4.2–6.1 × 2–2.3 μm and growing on dead bamboo.

##### Type.

China. Hainan, Haikou, Jinniuling Park, on dead bamboo, 18.XI.2020, Yu-Cheng Dai leg., *Dai 22116* (holotype BJFC036008).

##### Etymology.

*Bambusae* (Lat.): refers to the species growing on bamboo.

##### Fruiting body.

Basidiocarps annual, resupinate to effused-reflexed or pileate, separable from the substrate, without odour or taste and soft corky when fresh, corky and light in weight when dry. Pilei semicircular, projecting up to 1 cm, 1.5 cm wide and 5 mm thick at base; resupinate part up to 14 cm long, 6 cm wide and 2 mm thick at centre. Pileal surface bluish-grey when fresh, pale mouse-grey to greyish-sepia when dry, glabrous to slightly velutinate, rough, azonate; margin acute, incurved when dry. Pore surface bluish-grey to pale mouse-grey when fresh, becoming mouse-grey to dark grey when dry; sterile margin up to 1 mm wide; pores round to angular, 6–9 per mm; dissepiments thin, entire. Context white to cream, corky, up to 3.5 mm thick. Tubes paler than pore surface, corky, up to 1.5 mm long.

##### Hyphal structure.

Hyphal system dimitic; generative hyphae bearing clamp connections; skeletal hyphae IKI–, CB–; tissue unchanged in KOH.

##### Context.

Generative hyphae hyaline, thin- to slightly thick-walled, occasionally branched, 1.5–3 μm in diam.; skeletal hyphae dominant, hyaline, thick-walled with a narrow lumen to subsolid, occasionally branched, interwoven, 2–4.5 μm in diam.

##### Tubes.

Generative hyphae hyaline, thin- to slightly thick-walled, rarely branched, 1.5–2.5 μm in diam.; skeletal hyphae dominant, hyaline, thick-walled with a narrow lumen to subsolid, occasionally branched, flexuous, interwoven, 2–3 μm in diam. Cystidia absent; fusoid cystidioles present, hyaline, thin-walled, 11–18 × 2.5–4 μm. Basidia short clavate to barrel-shaped, bearing four sterigmata and a basal clamp connection, 13–19 × 4.5–5.5 μm; basidioles dominant, in shape similar to basidia, but smaller.

##### Spores.

Basidiospores cylindrical to oblong ellipsoid, hyaline, thin-walled, smooth, IKI–, CB–, (4–)4.2–6.1(–6.5) × (1.9–)2–2.3(–2.6) µm, L = 4.917 µm, W = 2.109 µm, Q = 2.26–2.41 (n = 90/3).

##### Type of rot.

Brown rot.

##### Additional specimens (paratypes) examined.

China. Hainan, Haikou, Jinniuling Park, on dead bamboo, 7.XI.2020, Yu-Cheng Dai leg., *Dai 21942* (BJFC035841), 18.XI.2020, *Dai 22104* (BJFC035996), *Dai 22110* (BJFC036002) and *Dai 22114* (BJFC036006).

**Figure 3.**
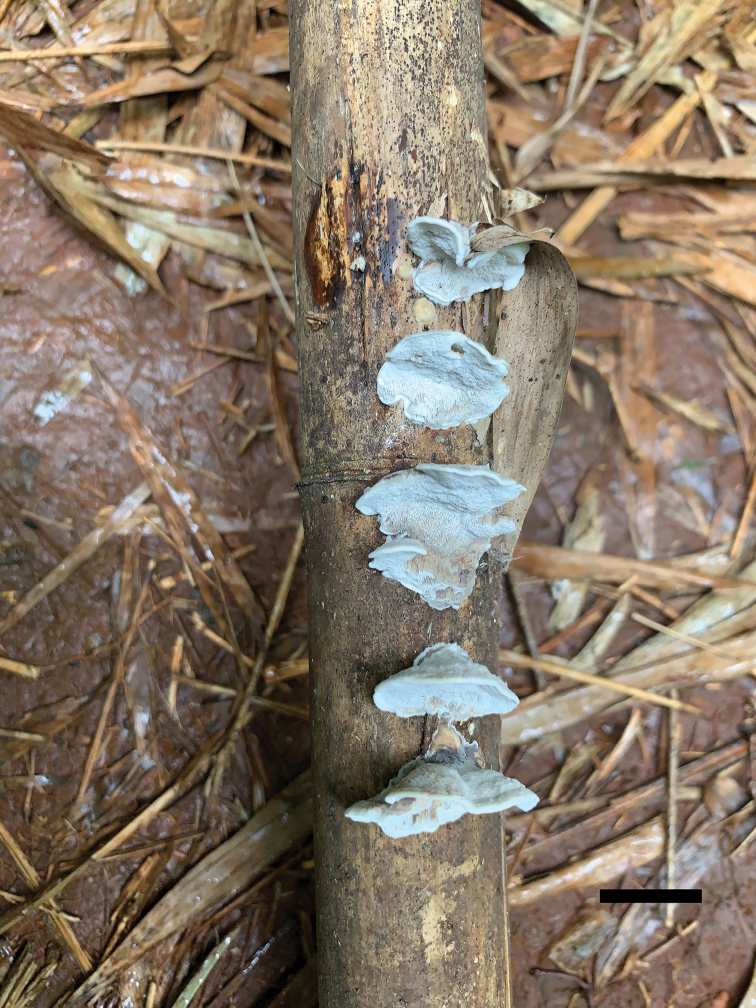
Basidiocarps of *Fomitopsisbambusae* (holotype Dai 22116). Scale bar: 1.0 cm.

**Figure 4.**
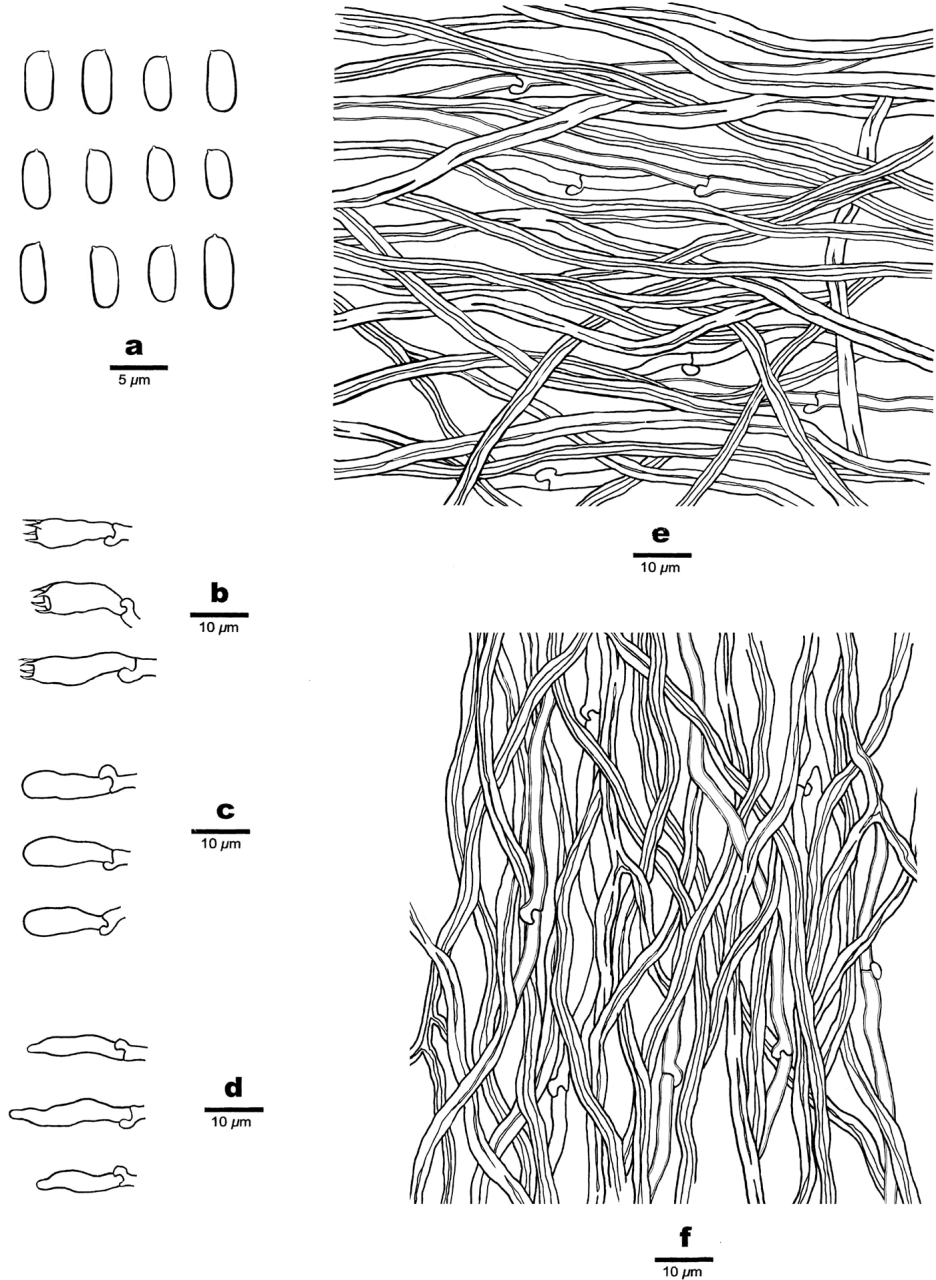
Microscopic structures of *Fomitopsisbambusae* (drawn from the holotype) **a** basidiospores **b** basidia **c** basidioles **d** cystidioles **e** hyphae from context **f** hyphae from trama.

**Table 4.** A comparison of species in the *Oligoporus*.

Species	Basidiocarps	Pore (per mm)	Pore surface	Cystidia	Cystidioles	Basidiospores size (μm)	Basidiospores shape	Reference
*** Oligoporus podocarpi ***	**Resupinate**	**Round to angular, 5–6**	**White to pale cream**	**Thick-walled with apically encrusted**	**Absent**	**3.8–4.2 × 2–2.5**	**Allantoid to oblong ellipsoid**	**Present study**
* O. rennyi *	Resupinate	Angular, 2–4	White or cream, then pale brown	Absent	Absent	4.8–6 × 2.5–3.5	Oblong ellipsoid	Ryvarden and Melo (2014); Shen et al. (2019)
* O. sericeomollis *	Resupinate	Round and angular, 4–6	White or discoloured yellowish or tan	Thick-walled with apically encrusted	Present, thin-walled	4–5 × 2–2.5	Oblong cylindrical to ellipsoid	Ryvarden and Melo (2014); Shen et al. (2019)

#### ﻿ Oligoporus podocarpi

Taxon classificationFungiPolyporalesPolyporaceae

Y.C. Dai, Chao G. Wang & Yuan Yuan sp. nov.

5209892F-1DF4-541D-98F8-F789BE9F9D3E

MycoBank No: 839360

Figs 5 , 6

##### Diagnosis.

*Oligoporuspodocarpi* is characterised by soft fresh basidiocarps, becoming rigid upon drying, a monomitic hyphal system with hyaline clamped generative hyphae, the presence of apically encrusted cystidia, broadly ellipsoid to reniform, dextrinoid, cyanophilous basidiospores measuring 3.8–4.2 × 2–2.3 μm, and growing on rotten wood of *Podocarpus*.

##### Type.

China. Hainan, Changjiang, Hainan Tropical Rainforest National Park, Bawangling, rotten wood of *Podocarpusimbricatus*, 10.XI.2020, Yu-Cheng Dai leg., *Dai 22042* (holotype BJFC035938).

##### Etymology.

*Podocarpi* (Lat.): referring to the species growing on wood of *Podocarpusimbricatus*.

##### Fruiting body.

Basidiocarps annual, resupinate, adnate, soft corky, with mushroom odour when fresh, becoming rigid when dry, mild taste, up to 3 cm long, 2 cm wide and 2.3 mm thick at the centre. Pore surface snow white when fresh, becoming cream to buff upon drying, somewhat glancing; sterile margin indistinct, thinning out, up to 0.3 mm wide; pores round to angular, 5–6 per mm; dissepiments thin, entire. Subiculum white, fibrous to soft corky when dry, up to 0.3 mm thick. Tubes concolorous with the pore surface, hard corky to brittle when dry, up to 2 mm long.

##### Hyphal structure.

Hyphal system monomitic; generative hyphae with clamp connections, smooth, hyaline, IKI–, CB–; tissues unchanged in KOH.

##### Subiculum.

Generative hyphae thick-walled with a wide lumen, occasionally branched, flexuous, interwoven, 2.5–3.8 μm in diam.

##### Tubes.

Generative hyphae thin- to thick-walled, occasionally branched, subparallel along the tubes to loosely interwoven, 2–3.1 μm in diam. Cystidia present, ventricose, very thick-walled, some apically encrusted. Basidia short clavate, sometimes with an intermediate constriction, with four sterigmata and a basal clamp connection, 12.5–16 × 4–5 μm; basidioles in shape similar to basidia, but smaller.

##### Spores.

Basidiospores broadly ellipsoid to reniform, hyaline, thin- to slightly thick-walled, smooth, often with one guttule, dextrinoid, CB+, (3.5–)3.8–4.2(–4.5) × 2–2.3(–2.5) µm, L = 3.98 μm, W = 2.14 μm, Q = 1.82–1.90 (n = 90/3).

##### Type of rot.

Brown rot.

##### Additional specimens (paratypes) examined.

China. Hainan, Changjiang, Hainan Tropical Rainforest National Park, Bawangling; rotten wood of *Podocarpusimbricatus*, 10.XI.2020, Yu-Cheng Dai leg., *Dai 22043* (BJFC035939) and *Dai 22044* (BJFC035940).

**Figure 5.**
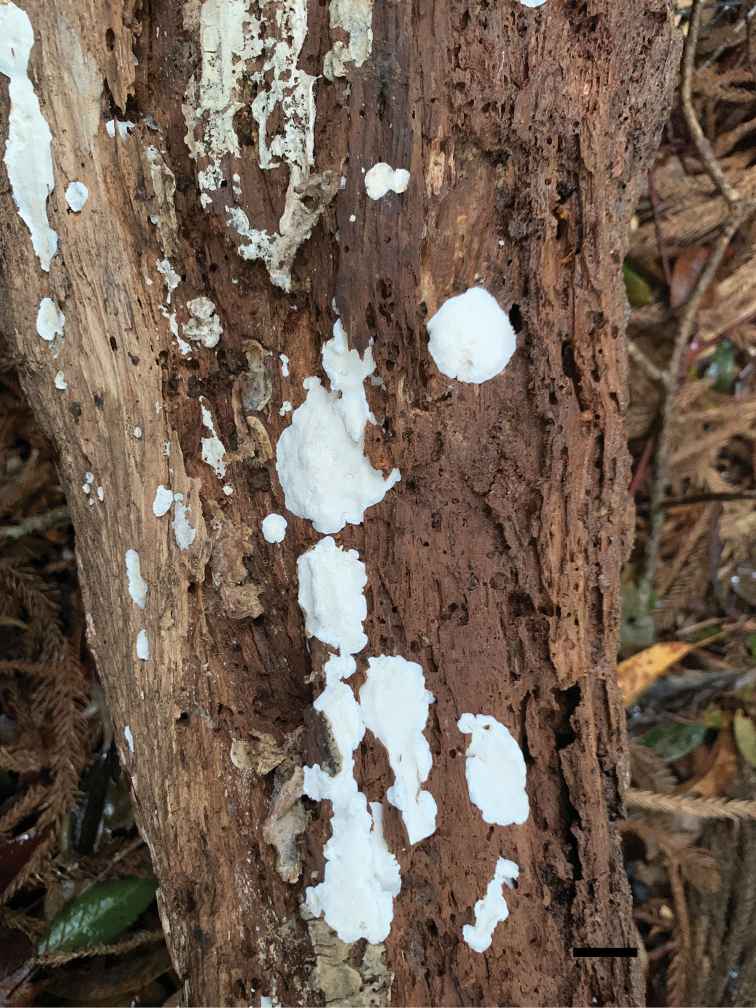
Basidiocarps of *Oligoporuspodocarpi* (holotype Dai 22042). Scale bar: 1.0 cm.

**Figure 6.**
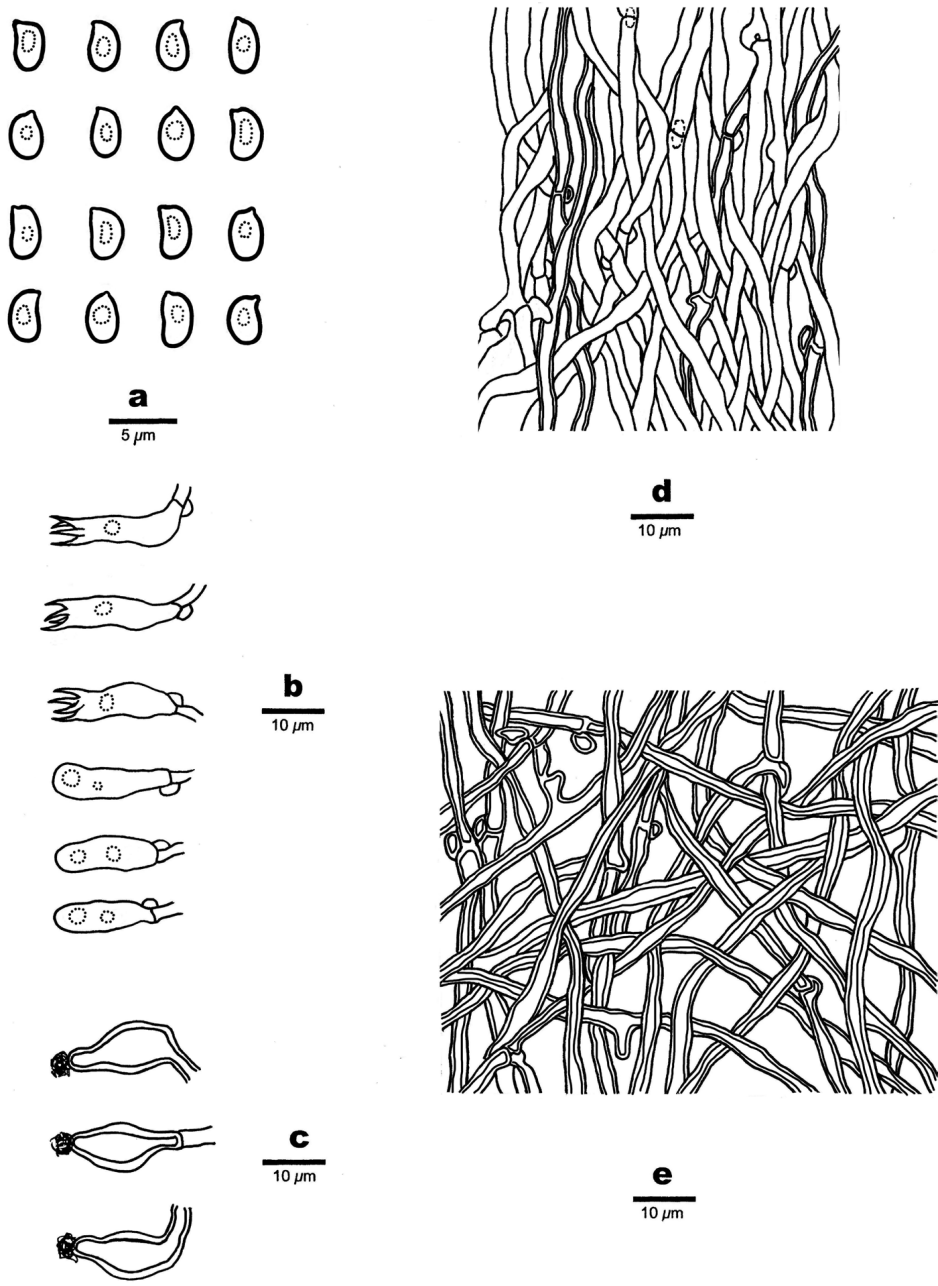
Microscopic structures of *Oligoporuspodocarpi* (drawn from the holotype) **a** basidiospores **b** Basidia and basidioles **c** cystidia **d** hyphae from subiculum **e** hyphae from trama.

## ﻿Discussion

In this study, two new species, *Fomitopsisbambusae* and *Oligoporuspodocarpi*, are described, based on morphological features and molecular data. The phylogenetic analysis of *Fomitopsis* (Fig. 1), inferred from ITS+nLSU+nuSSU+mtSSU+PRB2+TEF1 sequences, provides strong support (100% ML, 100% MP, 1.00 BPPs) for the placement of *F.bambusae* in *Fomitopsis* s.s. Besides, *Fomitopsisbambusae* formed a distinct and independent lineage, which is clearly distinguishable phylogenetically from all other known species of the genus. *Fomitopsisroseoalba* A.M.S. Soares and *F.subtropica* B.K. Cui & Hai J. Li are potentially the most closely related. Meanwhile, *F.roseoalba* is distinguished from *F.bambusae* by its larger pores (4–6 per mm vs. 6–9 per mm) and smaller basidiospores (3–4.9 × 1.8–2 µm vs. 4.2–6.1 × 2–2.3 µm, Tibpromma et al. 2017); *F.subtropica* is different from *F.bambusae* by smaller basidiospores (3.2–4 × 1.8–2.1 µm vs. 4.2–6.1 × 2–2.3 µm, Li et al. 2013).

Morphologically, *Fomitopsisbambusae*, *F.cana* (Blume & T. Nees) Imazeki, *F.caribensis*, *F.hemitephra* (Berk.) G. Cunn. and *F.nivosa* (Berk.) Gilb. & Ryvarden share approximately the same-sized pores (6–9 per mm). However, *Fomitopsiscana* differs from *F.bambusae* by its trimitic hyphal system, slightly larger basidiospores (5–6.2 × 2.1–3 μm, L = 5.81 μm, W = 2.6 μm vs. 4.2–6.1 × 2–2.3 µm, L = 4.917 µm, W = 2.109 µm) and grows on angiosperm wood rather than bamboo (Li et al. 2013). *Fomitopsiscaribensis* differs from *F.bambusae* by larger basidiospores (6–7.5 × 2.3–3.1 µm vs. 4.2–6.1 × 2–2.3 µm, Liu et al. 2019). *Fomitopsishemitephra* is distinguished from *F.bambusae* by its perennial habitat, woody hard basidiocarps (Cunningham 1965). *Fomitopsisnivosa* differs from *F.bambusae* by having longer basidiospores (6–9 × 2–3 µm vs. 4.2–6.1 ×2–2.3 µm, Gilbertson and Ryvarden 1986). In addition, *Fomitopsisbambusae* may be confused with *F.ostreiformis* (Berk.) T. Hatt. in having similar-sized basidiospores and also growing on bamboo, but *F.ostreiformis* differs from *F.bambusae* by the larger pores (3–4 per mm vs. 6–9 per mm) and trimitic hyphal system (De 1981).

Our phylogeny of *Oligoporus* (Fig. 2), based on ITS+nLSU+nuSSU+mtSSU+PRB1+PRB2+TEF1 sequence, demonstrated *Oligoporus* s.s. formed a monophyletic lineage with a robust rating (100% ML, 100% MP, 1.00 BPPs), which is distant from *Postia* s.s. Though *Oligoporus* and *Postia* are similar to each other in morphological characteristics, some significant differences remain. For instance, *Postia* s.s. has effuse-reflexed to pileate basidiocarps, thin-walled and acyanophilous basidiospores (Donk 1971; Ryvarden and Melo 2014; Shen et al. 2019), while *Oligoporus* s.s. has resupinate basidiocarps, slightly thick-walled and cyanophilous basidiospores (Shen et al. 2019). Phylogenetically, *Oligoporuspodocarpi* is nested in the *Oligoporus* s.s. clade with a strong support (100% ML, 100% MP, 1.00 BPPs) and related to *O.rennyi* (Berk. & Broome) Donk and *O.sericeomollis* (Romell) Bondartseva (Fig. 2). These three species, representing *Oligoporus* s.s., have resupinate basidiocarps, white to cream pore surface and thick-walled, dextrinoid, cyanophilous basidiospores. However, *Oligoporusrennyi* differs from *O.podocarpi* in the very fragile dry basidiocarps, the lack of cystidia and the presence of chlamydospores (Donk 1971; Ryvarden and Melo 2014). *Oligoporussericeomollis* is different from *O.podocarpi* by fragile dry basidiocarps, longer basidiospores (4–5 × 2–2.5 μm vs. 3.8–4.2 × 2–2.3 µm) and the extremely bitter taste (Núñez and Ryvarden 2001; Ryvarden and Melo 2014). Mophologically, *Oligoporuspodocarpi* is similar to *Postiasimanii* (Pilát) Jülich, *Cystidiopostiahibernica* (Berk. & Broome) B.K. Cui, L.L. Shen & Y.C. Dai and *Rhodoniarancida* (Bres.) B.K. Cui, L.L. Shen & Y.C. Dai by resupinate basidiocarps, white to cream pore surface (Jülich 1982; Núñez and Ryvarden 2001; Ryvarden and Melo 2014; Shen et al. 2019). However, *Postiasimanii* has smaller pores (6–8 per mm) and allantoid, thin-walled basidiospores measuring 4–5.3 × 0.8–1.2 µm (Jülich 1982; Ryvarden and Melo 2014). *Cystidiopostiahibernica* and *Rhodoniarancida* are different from *Oligoporuspodocarpi* by larger pores (2–3 per mm in *C.hibernica*, 2–4 per mm in *R.rancida*) and allantoid, thin-walled basidiospores (4.3–6 × 1.4–1.9 µm in *C.hibernica*, 5–7 × 2–2.5 µm in *R.rancida*) (Ryvarden and Melo 2014; Shen et al. 2019).

## Supplementary Material

XML Treatment for Fomitopsis bambusae

XML Treatment for Oligoporus podocarpi
